# Rapid Prediction of a Liquid Structure from a Single Molecular Configuration Using Deep Learning

**DOI:** 10.1021/acs.jcim.3c00472

**Published:** 2023-06-12

**Authors:** Chunhui Li, Benjamin Gilbert, Steven Farrell, Piotr Zarzycki

**Affiliations:** † Energy Geosciences Division, Lawrence Berkeley National Laboratory, 1 Cyclotron Road, Berkeley, California 94720, United States; ‡ NERSC, Lawrence Berkeley National Laboratory, 1 Cyclotron Road, Berkeley, California 94720, United States

## Abstract

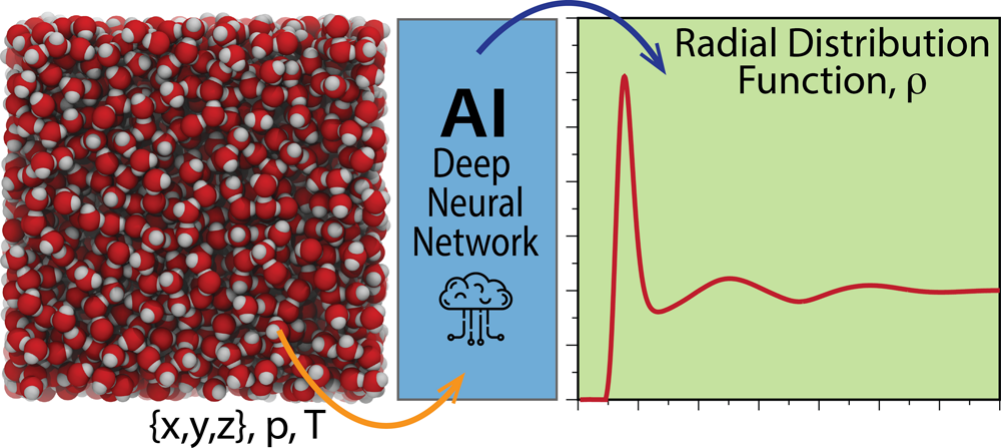

Molecular dynamics simulation is an indispensable tool for understanding the collective behavior of atoms and molecules and the phases they form. Statistical mechanics provides accurate routes for predicting macroscopic properties as time-averages over visited molecular configurations - microstates. However, to obtain convergence, we need a sufficiently long record of visited microstates, which translates to the high-computational cost of the molecular simulations. In this work, we show how to use a point cloud-based deep learning strategy to rapidly predict the structural properties of liquids from a single molecular configuration. We tested our approach using three homogeneous liquids with progressively more complex entities and interactions: Ar, NO, and H_2_O under varying pressure and temperature conditions within the liquid state domain. Our deep neural network architecture allows rapid insight into the liquid structure, here probed by the radial distribution function, and can be used with molecular/atomistic configurations generated by either simulation, first-principle, or experimental methods.

## Introduction

Molecular Dynamics (MD) simulation enables quantitative studies of the behavior of molecular systems, especially condensed phases in a broad range of disciplines.^1−3^ In the MD simulation, we compute forces acting on each entity and translate them to incremental displacements for a given time step via the integration algorithms that mimic the Newton equation of motion. The primary output of the molecular simulation is the simulation trajectory, which is a history of the atom positions, velocities, and forces in time.

The structural and dynamic properties of the molecular systems are estimated by analyzing the simulation trajectory and utilizing the statistical mechanics’ expressions linking molecular-level information with the macroscopic behavior.

In the case of liquids, the local structure, as probed by the pair correlation functions, is of fundamental importance. Among pair correlation functions, the radial distribution function (RDF) is most frequently discussed because it is accessible experimentally (X-ray and neutron scattering) and directly linked to the physicochemical properties of the liquid state. For example, in isothermal compressibility, the potential of mean force, total/potential energy, virial coefficients, or pressure can be expressed as a function of the RDF.^4^ The chemical potentials and osmotic pressure can also be expressed via the integrated RDF using the Kirkwood-Buff solvation theory.^5^ Finally, the interaction potential itself can be inferred from the RDF via reversed Ornstein–Zernike^6^ or reverse Kirkwood-Buff theories.^7^

The RDF is a generic joint probability function of finding one particle at the origin and another at some distance from the origin.^4^ It is obtained from the simulation trajectory as time or ensemble average and requires extensive simulations to cover a sufficient number of configurations to generate meaningful statistics. Unfortunately, long simulations are computationally expensive and not always feasible to carry out. For example, the most accurate quantum chemistry methods, such as coupled-cluster or multireference methods, are still computationally prohibitive to produce more than a single configuration.

MD simulation can produce substantial MD data that stores rich information to describe the state of the system. Depending on the needs of the researchers, the raw output of an MD simulation may include information on atomic identity, position, velocity, force, etc.

Molecular simulations can generate large data sets that can be explored using AI/ML methods. Indeed, utilizing AI/ML to surrogate or accelerate molecular simulations, expand their capabilities in terms of the interaction potential and time-step/simulation length, or overcome slow/energetically forbidden states is one of the most active domains in computational chemistry.

For example, deep neural networks (DNNs) have been trained on highly accurate quantum chemistry methods to improve descriptions of interactions.^8^ The long-standing issue of the high computational cost of quantum chemistry methods can be resolved using deep learning algorithms to construct the force field or estimate the energy surface.^9−18^ Many successful DNNs have been derived in this category, and the corresponding frameworks have been made available.^19−22^ Despite a noticeable increase in computational performance over conventional ab-initio computations, this approach is still less efficient than the classical MD simulation, especially for big complex systems. Besides, DNN methods have also been used to learn the dynamics of systems and accelerate simulations by replacing a traditional MD integrator.^23,24^ The DL-based integrator trained on MD trajectories enables a large time step without losing computing stability. However, this DL-based integrator is still limited by the size and complexity of the simulated system. Lastly, in addition to the high computational cost of running long-term MD simulations, analyzing the results of MD trajectories is a computational burden. Therefore, many efforts have been devoted to rapidly predict molecular/macroscopic properties by sampling or extracting important features from the output of MD trajectories.^25−29^ For most of these types of work, intensive preprocessing of the MD data or feature engineering work to select proper input features is frequently necessary, which also requires a lot of effort in feature selection and relies on the researcher’s domain expertise.

In this study, we proposed a DNN model using the raw output of an MD trajectory to predict the local liquid structure, as probed by the RDF, with minimal effort in the feature selection process. Specifically, we aimed to enable fast prediction of the RDF from a single MD configuration. The single MD configuration is extracted from a short MD trajectory; thus, performing averages over the long MD trajectory can be avoided. We illustrated our end-to-end RDF-estimator with increasing molecular and interaction complexity for three liquids. However, our approach can be easily expanded to other systems, including mixtures or heterogeneous phases.

## Methods

### Model Architecture

**Figure 1:**
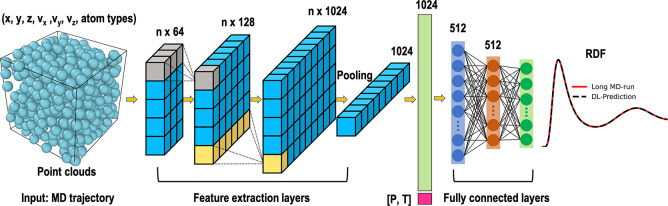
A schematic representation of PointNet-MD.

MD trajectories record the temporal evolution of atom movements during the simulation. It can be viewed as a series of sequential snapshots of a simulated molecular system. Each snapshot represents atomic coordinates at a specific time. Every atom in the system can be considered as a point with a corresponding coordinate (*x*_*i*_, *y*_*i*_, *z*_*i*_). Hence, every snapshot from an MD trajectory is a 3-dimensional point cloud with *N* points. *N* is the total number of atoms in the system. Therefore, the raw output of the MD trajectory can be treated as a series of point clouds. In this representation, the atomic bonds are not taken into consideration. Instead of extracting fingerprints from the complex MD data, we aim to develop an end-to-end deep learning model that directly uses the intricate MD data as input with the least amount of handcrafted work. Thus, we prefer a deep learning architecture that avoids the need for feature engineering that preprocesses the raw output from the MD simulation. At the moment, PointNet^30^ is one of the most frequently used deep learning models for learning features from 3-dimensional point cloud data and widely used for object detection and segmentation in automotive applications. In this work, we construct PointNet-MD inspired by the original PointNet architecture. PointNet-MD consists of three major components: convolutional layers, pooling, and fully connected layers. First, the position of all atoms and corresponding velocities and atom types are used as initial inputs. The convolution layers learn features from the initial inputs. Then, the pooling layer collects the most crucial features. Next, an additional scalar feature describes the simulation’s thermodynamic states and is combined with the features extracted from the previous step. Finally, the fully connected layers were used to analyze the combined features further to produce a prediction of the radial distribution function. A detailed introduction of inputs and outputs is explained in the next section. In this way, the single MD configuration can be mapped to the liquid local structure using the PointNet-MD model. Figure 1 displays a schematic representation of one PointNet-MD used in this paper.

All PointNet-MD models were employed in PyTorch^31^ and trained on a single NVIDIA A100 GPU from NERSC Perlmutter. The model is trained to minimize the loss function (MSE) between the long-run averaged RDF and DL-predicted RDF. During the training, the model uses Adam Optimizer^32^ with default parameters and the ReLU nonlinear activation function in each layer. The learning rate was set to 0.001. During the training, the Early Stopping strategy is applied to reduce overfitting. The number of epochs was initially set to 1000 but eventually determined by the Early Stopping strategy during the training process.

### Input and Output

PointNet-MD takes the single snapshot MD data (a set of point clouds) comprised of *N* atoms as input. Each point represents an atom in the MD simulation and includes the basic information dumped from MD simulations: atomic positions (*x*_*i*_, *y*_*i*_, *z*_*i*_), velocities, and atom types. Atomic positions are mandatory messages passed into neural networks. Thus, the input can be described as 3 + *M* features, where *M* is the number of additional features (atomic velocities, atom types, etc.) that the user wants to append to the atomic positions of each atom. For nonmonoatomic systems, we encoded atomic identity using one-hot encoding. For example, oxygen and hydrogen atoms can be encoded as  and , respectively. This type of encoding scheme can easily be extended to a more complex system consisting of more types of atoms. Each system’s pressure and temperature are considered in the middle of the neural network. A complete representation of input features used in this work is depicted in Figure 1. The input is a matrix of MD configuration with a dimension of (N, 3+M). The output is the distribution of the RDF which is represented as a fixed array of points, and the dimension of the output array depends on the systems that the model wants to predict.

### Data Set Preparation

#### MD Simulation

MD simulation is the first step in producing the data set required to build the PointNet-MD model. Deep learning can dig through a large volume of data to automatically identify patterns and extract features from complex data without human input. Consequently, a large number of samples from the data set are needed to train deep learning models effectively. The deep learning model’s accuracy depends on both the quantity and quality of the data. In this work, we performed extensive long MD simulations under various thermodynamic states. Table 1 lists the range of thermodynamic states and the total number of MD simulations that have been performed for each liquid system. To thoroughly investigate thermodynamic states in the liquid phase, we uniformly sampled pressure and temperature values from the liquid phase. In addition, the complexity of the system is increased by gradually adding more atoms to the solute molecule. In the end, we investigated three types of liquids: monatomic, diatomic, and triatomic systems, ranging from simple to complex.

**Table 1:** Range of Pressure and Temperature Values and the Total Number of MD Simulations Explored for a Given Liquid

Liquid	Pressure [atm]	Temperature [K]	no. of MD simulations
Ar	1–21	85–110	400
NO	30–70	120–160	200
H_2_O	1–217	273–373	110

All MD simulations were carried out using the Large-scale Atomic/Molecular Massively Parallel Simulator (LAMMPS)^33^ and visualized using OVTIO.^34^ The initial MD configurations for all simulations were prepared via the PACKMOL package.^35^ For each simulation, after minimizing the system, we run sequences of 500 ps heating (NVT ensemble) followed by density optimization (NPT ensemble) to reach the target temperature and pressure. Finally, the production simulations were conducted for 10 ns in the NPT ensemble.

#### Monatomic Liquid System

Liquid argon (Ar) is a prototypical monatomic system and a benchmark for simple fluids. It solely has Lennard-Jones potential, making it an excellent starting point for testing our PointNet-MD model. The simulation box size is 3 × 3 × 3 nm^3^ and contains 568 Ar atoms. 400 simulations are performed under the thermodynamic states listed in Table 1.

#### Diatomic Liquid System

Nitric oxide (NO) is one of a living cell’s most important signal molecules and plays an important role in physiological processes. Here, we use NO as a showcase of the diatomic liquid system. The compass force field was used to model the liquid NO.^36^ The simulation box is 3 × 3 × 3 nm^3^ and contains 1376 atoms. 200 simulations are performed under the thermodynamic states listed in Table 1.

#### Triatomic Liquid System

For triatomic liquids, water is ubiquitous on earth and shows rich behaviors in various research fields. Therefore, we choose water—the most widely studied and discussed complex fluid. In this study, the SPC/E model is used. The simulation box is 3 × 3 × 3 nm^3^ and contains 1340 water molecules in total. 110 simulations are performed under the thermodynamic states listed in Table 1.

#### Data Generation

As explained in the previous section, many MD simulations were conducted as the first step under a wide range of thermodynamic conditions in the liquid phase. After the production run, we sampled the raw MD data every 1 ps. Subsequently, the generated data can represent comprehensive states of the liquid. In this work, we aim to use the single MD configuration sampled from short-time MD trajectory data to predict the RDFs that require long-time MD trajectories. Hence, we only sample the MD snapshots from the first 2 ns from the MD trajectory. More specifically, the data sets used in this study were generated by splitting the first 2 ns MD trajectories into individual frames (also called snapshots). In the end, we sampled 1500 frames for Ar, 2000 frames for NO, and water from the simulation trajectory under each thermodynamic condition. Ultimately, we obtained 600,000 configurations for liquid Ar, 400,000 for liquid NO, and 22,0000 for liquid water. Each sampled frame stores information about atomic coordination, velocities, and atom types and is used as the input for PointNet-MD. The temporal-averaged RDF is used as the label for the training. The ground truth RDF (*g*_*ref*_(*r*)) is calculated by averaging the RDF temporally throughout the whole MD trajectory using the expression
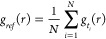
1where  is the RDF obtained from a single snapshot at the time *t*_*i*_, and *N* is the total number of snapshots sampled from the MD trajectory over 10 ns. We randomly split the data set into training, validation, and testing subsets based on (*P*, *T*) conditions. Specifically, 80% (*P*, *T*) of the conditions are in the training set, 10% (*P*, *T*) of the conditions are in the validation set, and 10% (*P*, *T*) of the conditions are in the test set.

## Results and Discussion

### Simple Fluid

**Figure 2:**
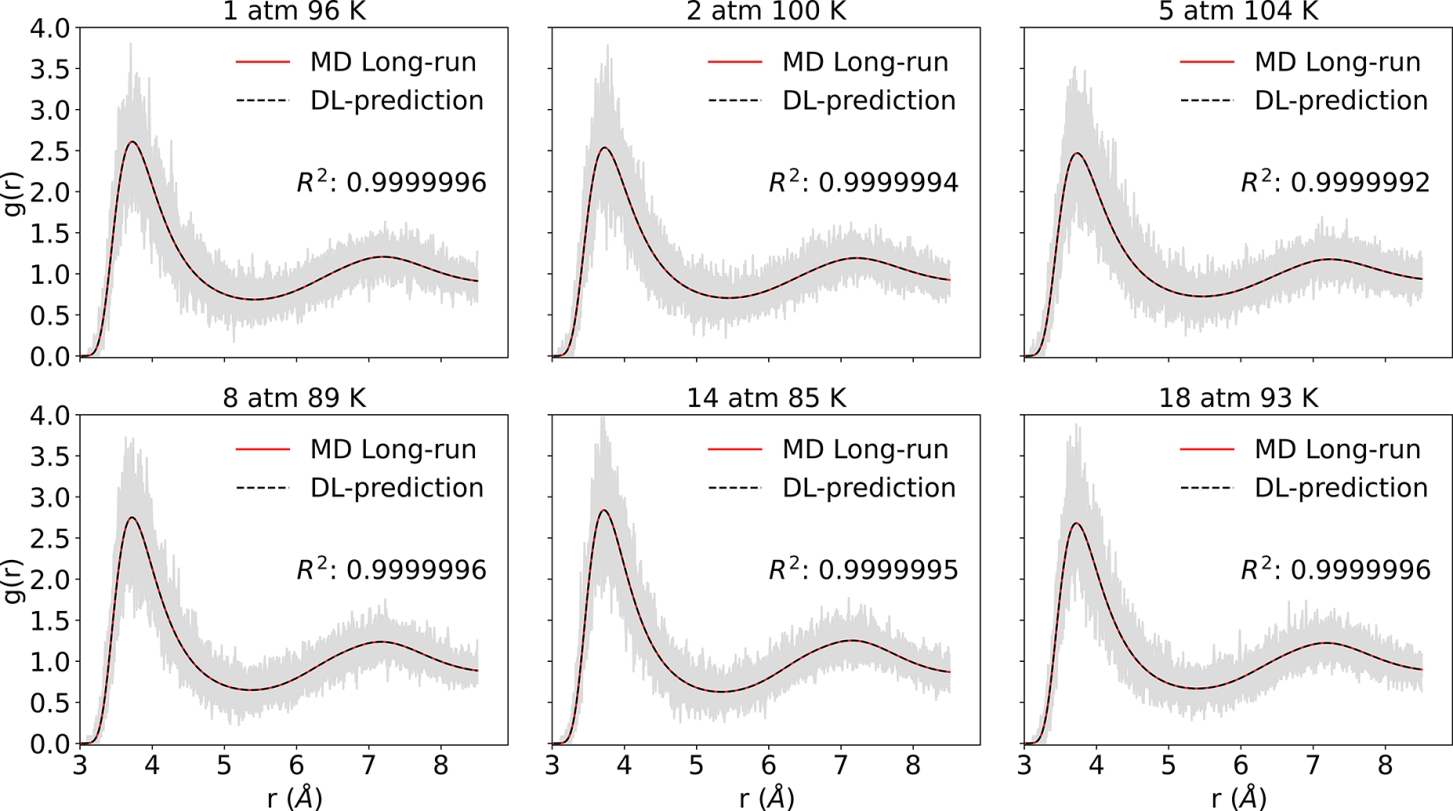
Comparison between the PointNet-MD prediction from one molecular configuration and the temporally averaged RDF for simple fluid Ar under six randomly selected thermodynamics states. Solid red lines show the temporally averaged RDF, and dashed black lines show PointNet-MD prediction. The gray region shows fluctuations of single snapshot RDFs around the temporally averaged RDF. *R*^2^ refers to average scores over all tested frames in the corresponding thermodynamic state.

We first evaluated PointNet-MD’s performance in predicting the structure of simple Lennard-Jones (LJ) fluids (Ar). We trained PointNet-MD on 320 (*T*, *P*) thermodynamic combinations of Ar in the liquid phase and tested the model on 40 (*T*, *P*) thermodynamic combinations that were never exposed to the neural network during the training procedure. The atom types in this monatomic system are omitted since only one type of atom exists. Hence, in this scenario, the input to PointNet-MD is atomic positions and velocities dumped from MD trajectories. Therefore, the input dimension is (*N*, 6), and *N* is the system’s total number of Ar atoms. The output is the RDF of Ar–Ar. After training, we randomly selected six thermodynamical states from the test results and compared them with the long-run averaged simulation results. The comparisons are displayed in Figure 2. It shows a great match in peak values and locations for Ar–Ar pairs between PointNet-MD predictions and corresponding ground truth RDFs. Since we have tested 1500 frames for each system condition, we have also calculated the mean *R*^2^ scores for each thermodynamic state. All test conditions received remarkably high mean *R*^2^ scores, as listed in the figure. The high mean *R*^2^ scores on test sets demonstrate that the model can accurately predict the local structure of Ar–Ar using a single piece of MD configuration information.

**Figure 3:**
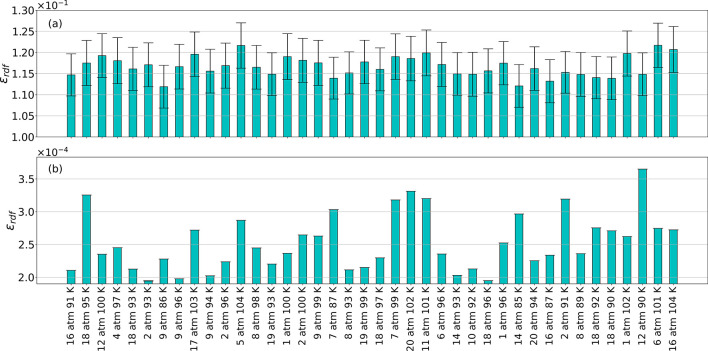
Error in estimating the RDF from a single molecular configuration. (a) Estimated RDF error from direct MD calculation and (b) estimated RDF error from PointNet-MD.

Moreover, we evaluated our model on two additional error metrics—MD estimation- and PointNet-MD prediction error from the single MD configuration. Both errors are determined using eq 2, where *g*_*pred*_(*r*) is an RDF estimated from either PointNet-MD or MD using one MD snapshot. Figure 3 exhibits the mean and standard deviation of the predicted errors as assessed by the aforementioned two schemes for all testing conditions in a bar plot. As shown, PointNet-MD’s prediction error is almost 3 orders of magnitude lower than the one derived from direct MD calculation. Also, the extremely low standard deviation from PointNet-MD indicates that all extracted frames from the same thermodynamic state have achieved highly accurate predictions. Hence, PointNet-MD can use a single MD frame extracted from this insufficient trajectory to predict the RDF curve that is identical to the one obtained from the long-MD trajectory.
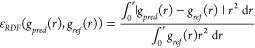
2

### Diatomic Simple Fluid

**Figure 4:**
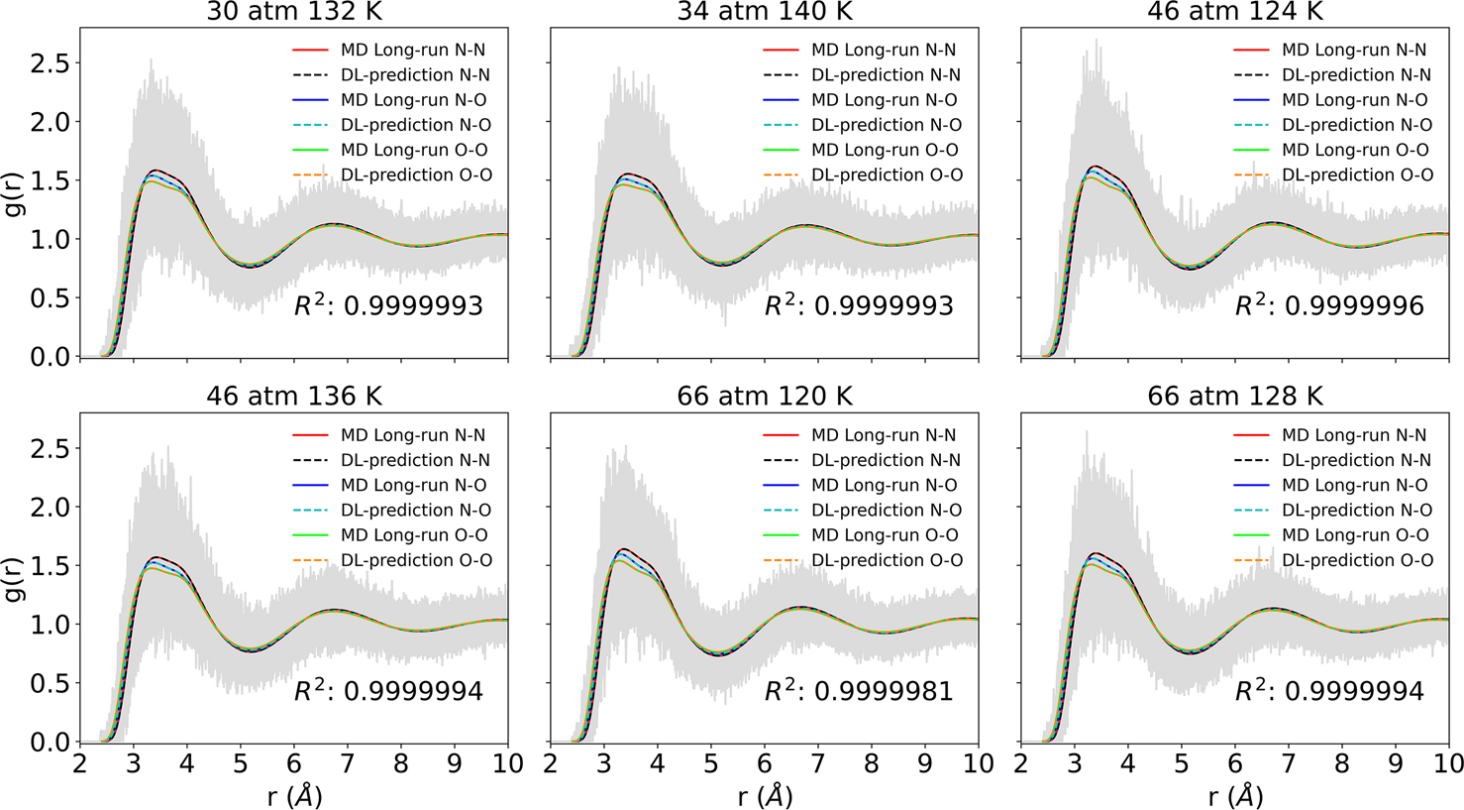
Comparison between PointNet-MD prediction from one molecular configuration and the temporally averaged RDF for liquid NO under six randomly selected thermodynamics states. Red, blue, and lime solid lines represent the temporally averaged RDF for N–N, N–O, and O–O pairs, respectively. Black, cyan, and orange dashed lines represent PointNet-MD predictions of N–N, N–O, and O–O pairs, respectively.

**Figure 5:**
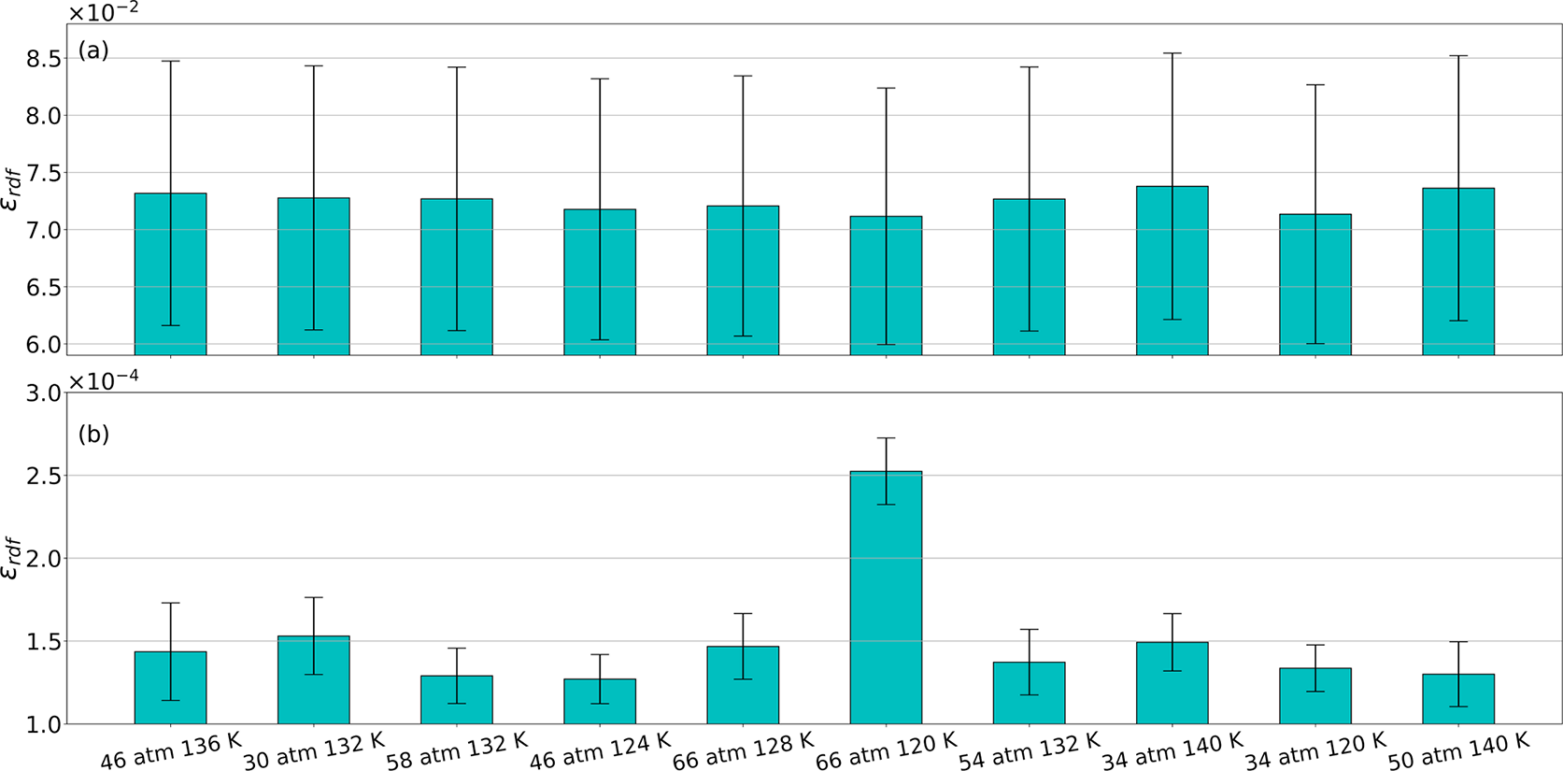
Error in estimating the RDF from a single molecular configuration for liquid NO. (a) Estimated RDF error from direct MD calculation and (b) estimated RDF error from PointNet-MD.

Liquid argon is a simple fluid with simple force interactions between atoms. Only short-range interactions are considered. To evaluate the performance of PointNet-MD for a more complicated system, we investigated the model’s performance on liquid NO. The NO system has three RDFs for the N–N, N–O, and O–O pairs, respectively. Similar to the monatomic system, we trained the model on 160 thermodynamic states and evaluated it on 20 additional thermodynamic states. In this case, atom types are encoded as [0, 1] (N) or [1, 0] (O). The encoded atom type is appended to the atomic positions and velocities as the input. The output is three RDFs for N–N, N–O, and O–O. Thus, with a single MD configuration provided, PointNet-MD will estimate all pairs simultaneously. Six randomly selected test results from the PointNet-MD prediction are illustrated in Figure 4. In the NO system, RDF curves of three atom pairs differ slightly in the peak values and corresponding peak locations. PointNet-MD is capable of recognizing the slight changes in RDFs for various pairs and predicts each RDF curve with high accuracy, as evidenced by the high mean *R*^2^ value. Still, Figure 5 presents the total mean and standard deviation of the prediction errors for all RDFs obtained from MD and PointNet-MD that used a single MD configuration. As shown in the figure, for each thermodynamic state tested, the overall estimation error of PointNet-MD is approximately 2 orders of magnitude smaller than the error calculated by MD.

**Figure 6:**
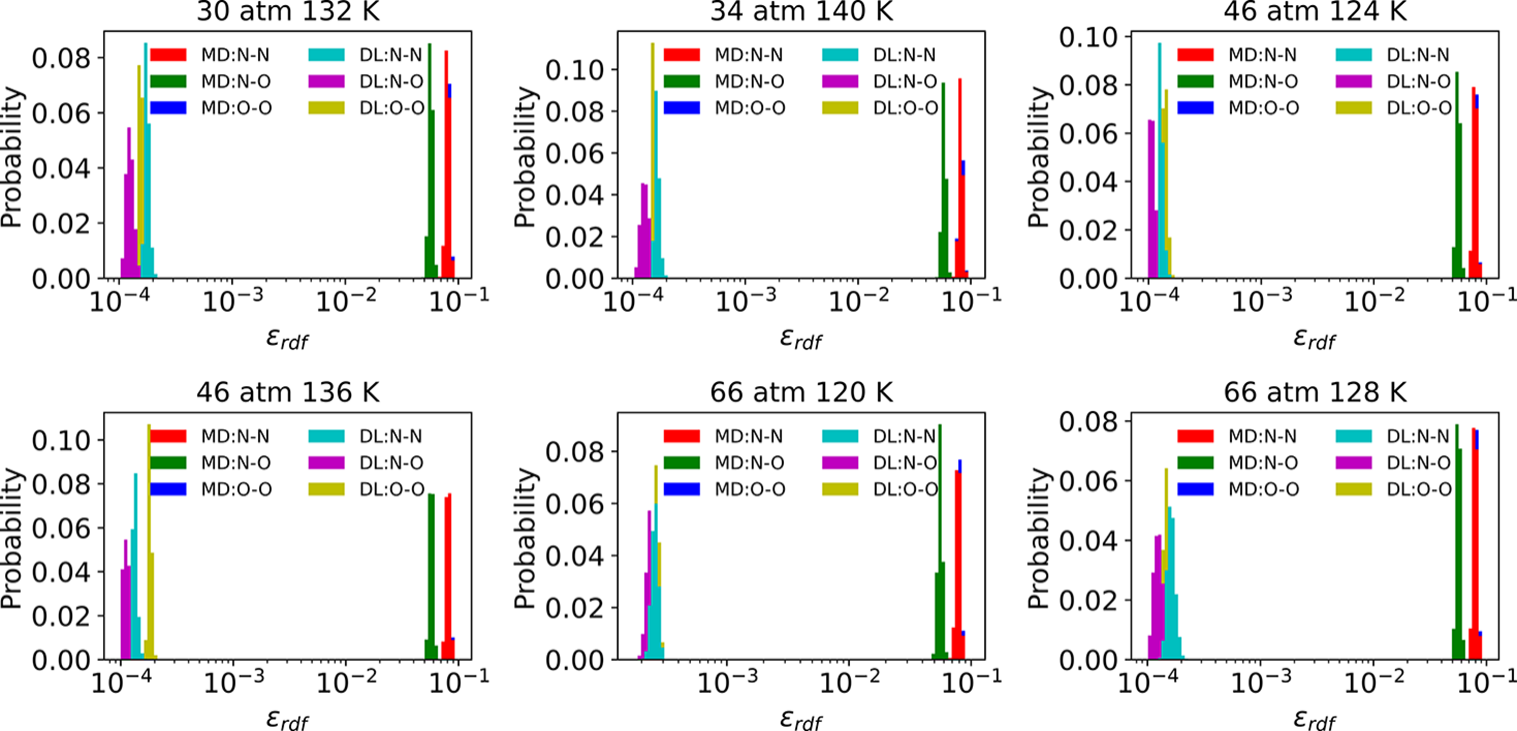
Comparison of each pair’s RDF prediction error distribution from direct MD calculation and PointNet-MD prediction under six thermodynamic states for liquid NO. Red, green, and blue denote the MD’s estimation error of N–N, N–O, and O–O pairs, respectively. Cyan, magenta, and yellow denote PointNet-MD’s estimation error of N–N, N–O, and O–O pairs, respectively.

Additionally, we calculated the error distribution for each atom pair. Figure 6 demonstrates the single RDF’s error distribution between MD and PointNet-MD for six selected tested cases. The figure suggests that the ε_*RDF*_(*g*_*pred*_(*r*), *g*_*ref*_(*r*)) for each pair obtained by MD and PointNet-MD is on the order of 10^–1^ and 10^–4^, respectively. Hence, for each pair, PointNet-MD’s prediction is approximately 3 orders of magnitude more accurate than MD’s prediction. Consequently, despite the increased complexity of the system compared to simple fluids, PointNet-MD consistently demonstrates excellent performance in predicting RDFs for all tested states. What is more, consistent with MD, PointNet-MD’s prediction errors for the N–N and O–O pairs are larger than that of the N–O pair.

### Complex Fluid

**Figure 7:**
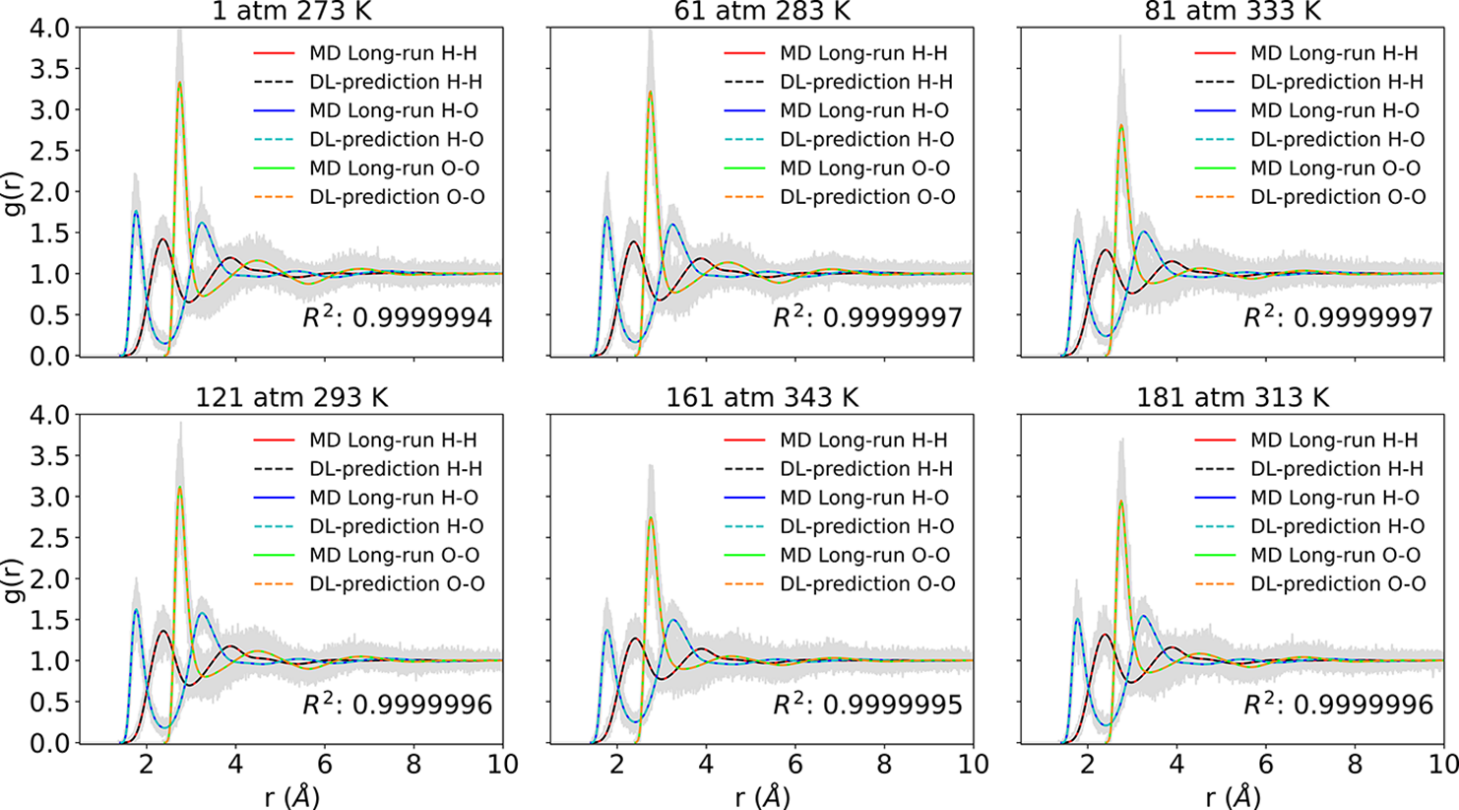
Comparison between PointNet-MD prediction from one molecular configuration and the temporally averaged RDF for water under six randomly selected thermodynamics states. Red, blue, and lime solid lines represent the temporally averaged RDF for H–H, H–O, and O–O pairs, respectively. Black, cyan, and orange dashed lines represent PointNet-MD predictions of H–H, H–O, and O–O pairs, respectively.

**Figure 8:**
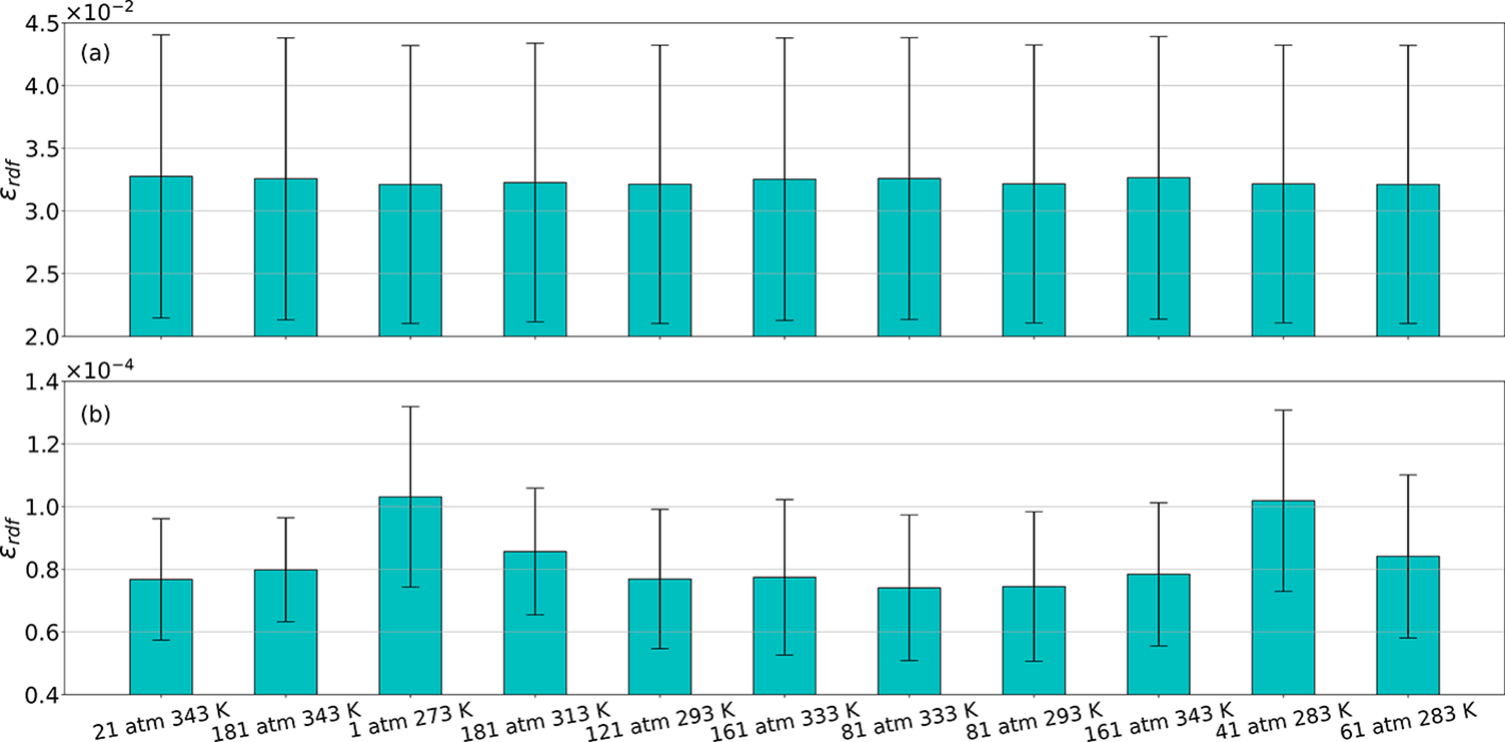
Error in estimating the RDF from a single molecular configuration for water. (a) Estimated RDF error from direct MD calculation and (b) estimated RDF error from PointNet-MD.

**Figure 9:**
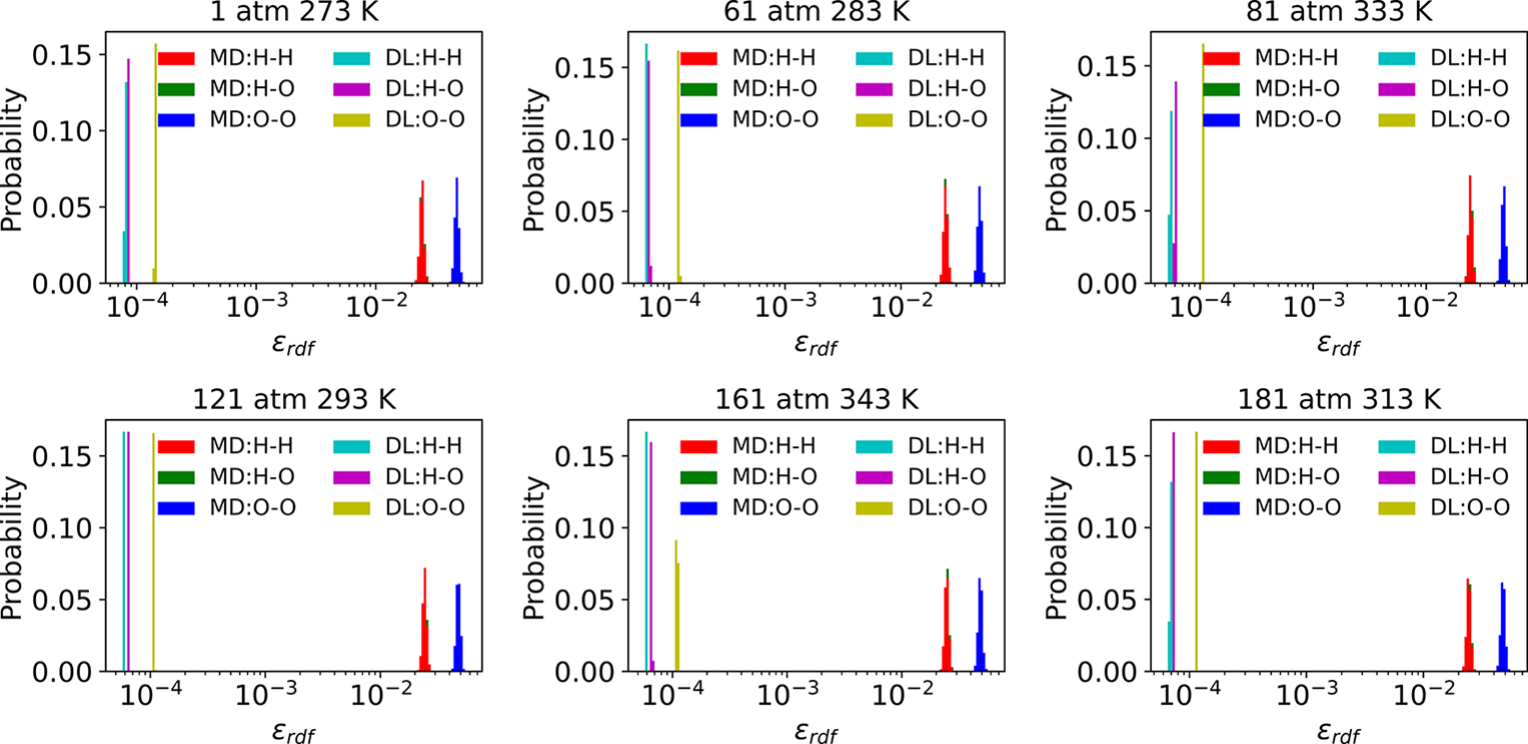
Comparison of each pair’s RDF prediction error distribution from direct MD calculation and PointNet-MD prediction under six thermodynamic states for water. Red, green, and blue denote the MD’s estimation error of H–H, H–O, and O–O pairs, respectively. Cyan, magenta, and yellow denote PointNet-MD’s estimation error of H–H, H–O, and O–O pairs, respectively.

Finally, we apply PointNet-MD to predict RDFs of pure water systems. Water contains three atoms of two types, H and O. Similar to NO, offering single MD framework information, PointNet-MD predicts RDFs for all H–H, H–O, and O–O pairs simultaneously. In contrast to the approximate peak locations in NO systems, each RDF in this scenario has distinct peak values and positions. Therefore, water systems are more complex to predict than the previous two liquids. Figure 7 lists six examples randomly sampled from the final prediction results on the test conditions. The figure illustrates that PointNet-MD could produce identical RDFs for all pairs as the MD long-time running average. In addition to correctly predicting the peak locations of distinct RDFs, PointNet-MD can also estimate the values of each pair’s peak. The error analysis in Figure 8 also reveals that by implementing PointNet-MD, the overall accuracy of estimating the RDF from a single MD frame can be improved by 2 orders of magnitude constantly. The error distribution of each RDF in Figure 9 clearly shows that the error of estimating *RDF*_*O*–*O*_ from MD and PointNet-MD is approximately 5 × 10^–2^ and 1 × 10^–4^, respectively, while the error of estimating *RDF*_*H*–*H*_ and *RDF*_*H*–*O*_ from MD and PointNet-MD is nearly 2.5 × 10^–2^ and 6 × 10^–5^, respectively. Consequently, the accuracy of predicting the *RDF*_*O*–*O*_, the *RDF*_*H*–*O*_, and the *RDF*_*O*–*O*_ can be improved by nearly 500 times through the use of PointNet-MD. In the estimation of all pairs using both methods, the *RDF*_*O*–*O*_ received the highest prediction error, and the *RDF*_*H*–*H*_ and the *RDF*_*H*–*O*_ gained the lowest prediction errors.

### Computational Efficiency

The average time for running a 10 ns MD simulation on the NERSC supercomputer is around 30 min for the argon system using 1 node, 1.5 h for the liquid NO system using 2 nodes, and 3 h for the water system using 2 nodes. While feeding a single snapshot of MD information into PointNet-MD, the prediction can be made in seconds. The prediction times for liquid Ar, NO, and water using a single MD configuration are 1.135, 1.219, and 2.08 s, respectively. Once that model was trained, the computational efficiency of estimating a reliable RDF of a new thermodynamics state using PointNet-MD has been significantly improved. The speed-up between PointNet-MD and MD simulation is up to 1000 orders of magnitude. Detailed speed-up information is listed in Table 2. As shown, with the increasing complexity of systems, the efficiency improvement is more notable without sacrificing any precision in prediction, as evidenced by the similar mean and standard deviation values of prediction errors among all systems. Table 2 also lists the model performances for all liquid cases on test sets, measured by an average *R*^2^ across all thermodynamic states for each liquid. Notably, high *R*^2^ scores are consistently observed for all liquid systems. Despite the gradual increase in system complexity, PointNet-MD has effectively maintained its capability to accurately predict RDFs.

**Table 2:** Accuracy and Efficiency Summary for Models^a^

System	Speed-up (×)	Metric	Test
Ar		*R*^2^	0.9999995
	1586	μ_*RE*_	0.000253
		σ_*RE*_	4.292e-05
NO		*R*^2^	0.9999993
	4430	μ_*RE*_	0.00015
		σ_*RE*_	4.039e-05
H_2_O		*R*^2^	0.9999996
	5192	μ_*RE*_	8.297e-05
		σ_*RE*_	2.558e-05

a *R*^2^ refers to the average scores in the test set. μ refers to the mean, and σ refers to the standard deviation. *RE* refers to relative errors between prediction and ground truth.

## Conclusion

We introduced an end-to-end deep learning model PointNet-MD derived from PointNet. PointNet-MD can directly process the MD configurations as a three-dimensional unordered point data cloud and accurately predict structural properties of liquids. Here, we showed that extensively trained PointNet-MD can predict time-averaged structural properties from a single-time frame configuration. In a traditional statistical mechanics approach, the time-average property is obtained by analyzing extensive simulation trajectory. In contrast, PointNet-MD can distinguish the most probable relative position of atoms from noise caused by thermal fluctuations and infer the equilibrium liquid structure from a single configuration.

In particular, we used PointNet-MD to predict the radial distribution function, one of the most important descriptors of the short-range liquid structure, and other physicochemical liquid properties (e.g., pressure, energy, compressibility, chemical potential). PointNet-MD was able to predict the RDF accurately for three types of liquids (Ar, NO, and H_2_O) that vary in molecular and interaction complexity.

In this work, we showed an example of the AI-driven ultrafast liquid structure prediction from limited simulation data. However, PointNet should be able to predict other statistical properties that are usually determined as time- or ensemble averages from simulation trajectory.

Having a set of AI-trained ultrafast property estimators, one can accelerate the chemical discovery in the high throughput framework relying on the computational screening of a candidate material/drug properties. The AI-trained predictors for the more complex systems with varying compositional/interaction complexity are yet to be developed. Our next step is to develop the interfacial properties of solvent next to the solid interfaces - a problem of paramount importance for electrochemistry, environmental chemistry, and catalysis.

## Data and Software Availability

The source code of the PointNet-MD model is available at https://github.com/nodameCL/PointNet-RDF-MDTraj. The training, validation, and test sets used in this work are available for download from https://zenodo.org/record/7776442.
